# Acrophialophora: A Comprehensive Review of Clinical Guidelines and Diagnosis

**DOI:** 10.7759/cureus.37614

**Published:** 2023-04-15

**Authors:** Abhijit Bhattaru, Isabella Blanchard, Sruthi Kunamneni, Chaitanya Rojulpote, Peter Iskander, Simin Nasr, Douglas Klamp

**Affiliations:** 1 Radiology, University of Pennsylvania, Philadelphia, USA; 2 Medicine, Rutgers University New Jersey Medical School, Newark, USA; 3 Biology, The College of New Jersey, Ewing, USA; 4 Internal Medicine, The Wright Center for Graduate Medical Education, Scranton, USA; 5 Nuclear Cardiology and Cardiovascular Molecular Imaging, University of Pennsylvania, Philadelphia, USA; 6 Family Medicine, The Wright Center for Graduate Medical Education, Scranton, USA; 7 Department of Internal Medicine, The Wright Center for Graduate Medical Education, Scranton, USA

**Keywords:** transplant, fungal keratitis, aids, pneumonia, lung infection, brain abscess, acrophialophora

## Abstract

*Acrophialophora* is a saprotrophic genus of fungi found in both temperate and tropical regions. The genus is comprised of 16 species, with the subspecies *A. fusispora *and *A. levis *necessitating the most clinical concern. *Acrophialophora* is an opportunistic pathogen with a broad range of clinical manifestations; the fungus* *has been implicated in cases of fungal keratitis, lung infection, and brain abscess. *Acrophialophora* infection is particularly of concern for immunocompromised patients, who often present with a more severe disease course involving disseminated infection and may not exhibit typical symptoms. Early diagnosis and therapeutic intervention are critical to the successful clinical management of *Acrophialophora *infection. Guidelines for antifungal treatment have yet to be established, partially due to the lack of documented cases. Aggressive use of antifungal agents and long-term treatment is required, especially in immunocompromised patients and patients with systemic involvement, due to the potential for morbidity and mortality. In addition to outlining the rarity and epidemiology of the disease, this review provides an overview of the diagnosis and clinical management of *Acrophialophora *infection to facilitate an early diagnosis and appropriate interventions.

## Introduction and background

Invasive fungal infections have risen in prominence over the past several years and are of concern for morbidity and mortality, especially in immunocompromised patients [1]. Most life-threatening incidences of fungal infection have occurred in highly immunocompromised patients, including patients with acquired immunodeficiency syndrome (AIDS), acute lymphoblastic leukemia (ALL), cystic fibrosis (CF), and transplant recipients [2].* A. fusispora* and *A. levis*, two subspecies of the genus *Acrophialophora*, are known opportunistic pathogens in humans; infection may manifest in a variety of ways, including fungal keratitis, lung infection, and brain abscess. 

Although *Acrophialophora* infection can be life-threatening, clinical disease identification and management have been limited due to the rarity of the condition and a lack of well-entrenched treatment guidelines. This review aims to provide an update on the clinical manifestations and management of *Acrophialophora* infection while briefly investigating the relevant epidemiology and rarity of the condition. Factors to consider when ruling out differentials will also be explained to facilitate a timely and accurate diagnosis of the *Acrophialophora* infection, which is critical to providing effective care and minimizing disease progression.

## Review

Rarity

*Acrophialophora* infections are rare; there are only 13 documented infections to date, with the first diagnosis occurring in 1981 (Table 1) [1-14]. Out of these, 10 were caused by *A. fusispora*, two were caused by *A. levis*, and one was caused by an unspecified *Acrophialophora* species. Additionally, there have been six documented cases of *Acrophialophora* colonization without signs of infection, largely in patients diagnosed with cystic fibrosis (CF) [4,5,8]. Some of these infections were confirmed as *Acrophialophora* by sequencing, but others were only identified morphologically, which could potentially mean that there were false positives [7]. It is likely, conversely, that *Acrophialophora* infections have been underdiagnosed due to the rarity of the fungus and confusion with similar species such as *Lomentospora prolificans*, *Scopulariopsis chartarum*, and *Paecilomyces spp.*, underscoring the importance of accurate identification [9,10].

**Table 1:** Instances of human Acrophialophora infection, clinical manifestation, and treatment outcomes Unk. = unknown, LAMB = liposomal amphotericin B, AMB = amphotericin B, ITRA = itraconazole, ALL = acute lymphoblastic leukemia, VRC = voriconazole, FLU = fluconazole HIV = human immunodeficiency virus, AIDS = acquired immunodeficiency syndrome, CAS = caspofungin, COPD = chronic obstructive pulmonary disease, MCTD = mixed connective tissue disease, CPA = chronic pulmonary aspergillosis. Instances of transient or chronic *Acrophialophra* airway colonization without infection are not shown.

Reference	Year of Diagnosis	Location	Species	Age/ Gender	Infection location	Medical history	Symptoms	Antifungal treatment	Outcome
Shukla et al. [3]	1981	India	A. fusispora	40/F	Eye	Fingernail trauma to the eye	Pain, redness, photophobia, lacrimation, white corneal lesion	Nystatin, potassium iodide	Improved, hypopyon disappeared, pain regressed, vision improved
Sutton et al. [5,11]	1997	United States	A. fusispora	Unk.	Pulmonary infection	Unk.	Unk.	Unk.	Unk.
Guarro et al. [11]	1997	Spain	A. fusispora	67/M	Lungs	Pulmonary fibrosis, emphysema, lung transplant	Asymptomatic, later pneumonia	LAMB, nebulized AMB, ITRA	Died
Al-Mohsen et al. [1]	1998	Saudi Arabia	A. fusispora	12/F	Lungs, brain	ALL, neutropenia	Fever, seizure	AMB, LAMB, ITRA	Improved clinically and radiologically
Arthur et al. [12]	Unk. (published 2001)	United States	A. fusispora	76/F	Left eye (keratouveitis)	Retained contact lens	Acute keratitis, lacrimation, discharges, eyelid swelling	Removal of necrotic mass	Improved, visual acuity increased
Guarro et al. [11]	2002	Portugal	A. fusispora	33/M	Lungs	Bilateral lung transplant	Unk.	VRC	Improved
Guarro et al. [5]	2002	Portugal	A. fusispora	Unk.	Eye (cornea)	Retained contact lens	Unk.	Unk.	Unk.
Guarro et al. [11]	2005	India	A. fusispora	55/F	Left eye	Wood chip injury	Pain, discharge, watering, redness, blurred vision, corneal ulcer	FLU	Therapeutic keratoplasty
Li et al. [13]	2010	Taiwan	A. fusispora	60/M	Brain and lungs	Pneumonia, corticosteroid use, HIV/AIDS, hepatitis	Fever, headache, altered mental status	VRC	Died of intra-cerebral hemmorhage
Watanabe et al. [5,14]	2017	Japan	Unspecified *Acrophialophora spp.*	77/M	Right eye (conjunctival ulcer, uveitis)	Neutropenia, prostate carcinoma, gardening hobby, history of travel	Congested and painful eye, blurred vision, partial abscess formation	ITRA, LAMB, VRC	Improved, vision was maintained (LAMB caused flare in symptoms)
Huang and Liu [7]	2018	China	A. levis	71/M	Lungs	Smoker, emphysema, multi-system organ failure	Fever, chills, dizziness, abdominal pain, cough, yellow purulent sputum, chest pain	CAS, LAMB	Initial improvement, died after LAMB discontinued
Samaddar et al. [5]	2019	India	A. fusispora	59/M	Lungs	COPD, MCTD, influenza A, CPA	Shortness of breath, cough, thick yellow sputum	ITRA	Improved clinically and radiologically
Modlin et al. [6]	Unk. (published 2020)	United States	A. levis	54/F	Brain (left thalamus)	Hypertension, type 2 diabetes, end-stage renal disease, organ transplant	Right-sided weakness, right-sided facial droop, decreased strength in extremities	VRC	Improved, mental status returned to baseline, right-sided weakness persisted
Sharma et al. [2]	Unk. (published 2022)	India	A. fusispora	26/M	Left eye (cornea)	No history of trauma, no comorbidities	Pain, watering, redness, blurred vision, whitish discoloration, corneal ulcer	VRC, natamycin, ITRA, AMB	Therapeutic keratoplasty

Epidemiology

*Acrophialophora* is a genus of heat-resistant, asexually reproducing soil fungi comprised of 16 species [15] and found in temperate and tropical climates [7]. Colonies are flat, between 30-60μm in diameter, initially white and later turning pale yellow or brownish gray with a dark reverse, and possess a velvety or felt-like texture [9]. 

Microscopically, visible differences exist between the two species. *A. fusispora* typically has slightly larger phialide and conidial sizes compared to *A. levis*. The conidial shape is ovoid to fusiform in *A. fusispora* but ellipsoid to cylindrical in *A. levis*. Conidial ornamentation in *A. fusispora* is finely echinulate to spiral sculpted, smooth to finely echinulate in *A. levis*. Conidial color is subhyaline to brown in *A. fusispora* and hyaline to subhyaline in *A. levis* [1,9]. 

*A. fusispora* and *A. levis* share 99.9% similarity in the large subunit ribosomal (LSU) region but differ in their internal transcribed spacer (ITS) and β-tubulin (Tub) sequences, with <96.1% and <96.6% sequence similarity, respectively [9]. *A. fusispora* has been mistaken for *L. prolificans* and other similar species, but in addition to molecular differences, distinctive morphological variations can help identify *A. fusispora* correctly. One unique feature is the conidial ornamentation, as described previously. Combined with the coloring and conidial shape, it is possible to distinguish *A. fusispora* from other species.

Instances of *Acrophialophora* infection have been found in Saudi Arabia, Sudan, India, Taiwan, China, Japan, Portugal, Spain, and the United States. Both males and females of various ages have been infected, with the youngest and oldest reported infected patients being 12 and 77 years old, respectively. *Acrophialophora* airway colonization has been seen in individuals as young as four years old [4]. Case studies of patients with *Acrophialophora* infections reveal several risk factors. Transplant patients are at higher risk of infection, and multiple instances of infection have been reported in lung and kidney transplant patients [6]. Other identified risk factors include HIV/AIDS, cancer, CF, keratitis, neutropenia, pneumonia, and the use of immunosuppressant drugs. Patients with CF are vulnerable to transitory or chronic airway colonization by *A. fusispora*, usually preceded by *Aspergillus fumigatus* colonization and typically not accompanied by bronchopulmonary episodes or evidence of tissue infection [4].

Disease manifestation

*Acrophialophora* has been implicated in occurrences of fungal keratitis, lung infection, and brain abscess, with symptoms varying widely across patients (Table 1). Most patients exhibit a milder disease course after being exposed to the pathogen through injury or trauma; infection in immunocompromised patients is more commonly promulgated throughout the body, especially in the lungs and central nervous system (CNS). *Acrophialophora* case reports indicate that lung infection is most common in immunocompetent patients, while immunocompromised patients form the majority of individuals with CNS involvement [5].

Eye Infection

Among reported cases, fungal keratitis due to *A. fusispora* infection is more common in immunocompetent patients than in immunocompromised patients. Most reported cases of *A. fusispora* ocular infection, especially in immunocompetent patients, are thought to be secondary to eye injury or the introduction of foreign substances into the eye. The first reported case of mycotic keratitis from an *A. fusispora* infection was reported in India and involved a 40-year-old patient who reported trauma from nails to her eye. Another female patient experienced keratouveitis due to *A. fusispora* infection promoted by prolonged contact use without removal [10,12]. 

Clinically, *Acrophialophora*-related fungal keratitis is characterized by pain, watering, redness, blurred vision, discharge, and discoloration in the infected eye [2]. Corneal ulcers, abscesses, or congested conjunctiva may also be present [14]. Although patients may recover quickly, instances of corneal perforation secondary to *A. fusispora* infection have been documented. A 27-year-old patient with blurred vision in one eye for 3-4 days reported pain, watering, and redness in his left eye, accompanied by a dry-looking corneal ulcer with infiltration and satellite lesions [2]. Treatment with both topical and systemic antifungal medication was unsuccessful, and therapeutic keratoplasty was performed after the patient’s vision continued to deteriorate and a perforated corneal ulcer formed.

Instances of *Acrophialophora* ophthalmologic infection have also been documented in immunocompromised patients [14]. A 77-year-old hemodialysis patient with neutropenia and prostate carcinoma reported that his right eye had been infected for two days; the patient’s conjunctiva was congested, forming a partial abscess. The patient was diagnosed with an *Acrophialophora spp*. infection after rRNA analysis. Although the patient did not report direct exposure or injury to the eye, she enjoyed gardening, indicating a possible soil exposure to *A. fusispora*. The patient’s symptoms resolved after management with systemic and topical voriconazole (VRC) and after unsuccessful treatment with liposomal amphotericin B (LAMB).

Pulmonary Infection

*Acrophialophora spp*. has been implicated in several cases of pulmonary infection. Patients with chronic obstructive pulmonary disease (COPD) may also be more prone to lung colonization and infection due to poor mucociliary clearance [5]. Clinical symptoms of pulmonary *Acrophialophora* infection include shortness of breath, a dry or productive cough, and thick sputum [5,13], which is often yellow and purulent and may be bloody [7].

Though many patients with *Acrophialophora*-related pulmonary infection eventually or concurrently exhibit cerebral infection, rapid diagnosis and aggressive treatment of pulmonary fungal infection may prevent disease progression, reducing morbidity and mortality. One case study involved a pulmonary fungal infection with no cerebral involvement in a 59-year-old male farmer with a history of pulmonary tuberculosis and COPD who was treated with steroids for mixed connective tissue disease [5]. A chest X-ray showed bilateral interstitial infiltrates, contrast-enhanced computed tomography of the patient’s chest suggested aspergilloma, and the serum* A. fumigatus* IgG level was elevated. The patient was ultimately diagnosed with influenza A with underlying chronic pulmonary aspergillosis. After treatment with oseltamivir for two weeks, the patient felt no relief. Internal transcribed spacer (ITS) gene analysis of the patient’s sputum specimen indicated an *A. fusispora* infection. The patient was treated with antifungal therapy and had clinical improvement after 10 days of treatment. Clinical and radiological improvement was noted after three months with no progression to the CNS.

CNS Infection

*Acrophialophora* has the potential to be neurotropic [1], with melanin in the cell wall likely serving as a virulence factor [6]. Although the pathogenesis of *Acrohpialophora* infection is unclear [7], how melanized fungi, including *Acrophialophora*, achieve cerebral infection is presumed to be either through the paranasal sinuses or secondary to pulmonary infection [6]. 

Symptoms of *Acrophialophora*-related brain abscess include fever, headache, and neurological deficits, including hemiplegia and seizures [1,6]. Many patients with *Acrophialophora* brain abscess have a history of prior or concurrent pulmonary fungal infection. Cerebral *Acrophialophora* infection is particularly common in immunocompromised individuals who may not exhibit typical symptoms [6]. The clinical management of immunocompromised patients must, therefore, include vigilant monitoring for opportunistic fungal infections, including *Acrophialophora*, as rapid diagnosis and treatment are necessary to prevent morbidity and mortality in this population.

In one case study involving *A. fusispora*, infection resulted in a fatal brain abscess in a 60-year-old male with AIDS [13]. The patient had a bitemporal headache and intermittent fever beginning two weeks before arrival at the hospital, as well as an altered level of consciousness upon arrival at the hospital. CT and MRI of the patient’s brain showed bilateral cerebellar lesions with mass effect as well as lesions in the left frontal lobe with irregular rim enhancement and ventriculitis. No meningeal enhancements or spinal lesions were reported. The presence of hyphae upon obtaining a specimen post-craniotomy was indicative of a fungal infection, and fungal growth on plates was identified as *A. fusispora* via microscopy; this was confirmed by DNA sequence analysis of the ITS1 and ITS2 genes. After arrival at the hospital and throughout antifungal disease management, the patient was diagnosed with a disseminated *Mycobacterium kansasii* infection, a GI bleed, ganciclovir-resistant cytomegalovirus, and hepatitis, which complicated treatment. This incident occurred several months after treatment for a non-resolving dry cough and a right lower-lung patch in his chest X-ray; no obvious pathogenic cause was identified for these symptoms, and the patient was diagnosed with cryptogenic organizing pneumonia and treated with corticosteroid therapy, highlighting the intersection between pulmonary and cerebral *Acrophialophora* infection.

Another immunocompromised patient being treated for acute lymphoblastic leukemia (ALL) exhibited *Acrophialophora* infection with both lung and CNS involvement [1]. A CT scan of the patient’s chest showed pulmonary nodules, and the patient was treated with amphotericin B (AMB) as antifungal therapy. After three weeks of treatment, the pulmonary nodules had progressed, and the patient suffered a seizure. A CT scan of the patient’s brain was abnormal. After another 26 days of LAMB treatment with continued intermittent fever, a CT and MRI of the patient’s brain showed an abscess. A culture of the drainage from the patient’s abscess revealed an *A. fusispora* infection, and the patient improved clinically and radiologically after adding itraconazole (ITRA) and increasing the patient’s LAMB and ITRA dosages. These results were consistent with later antifungal susceptibility testing of the isolate, in which the isolate exhibited susceptibility to ITRA and higher AMB concentrations (AMB MLC = 1.0 μg/ml at 24h and 48h, ITRA MLC = 0.125 μg/ml at 24h and 0.25 μg/ml at 48h). The addition of ITRA may also have positively affected the patient’s status due to ITRA’s favorable tissue distribution: brain tissue concentrations can significantly exceed analogous plasma levels [1,16].

*A. levis* has also been implicated in cerebral infections. One case study involved a 54-year-old female kidney transplant recipient with hypertension, type 2 diabetes, neutropenia, and end-stage renal disease [6]. Following transplant complications, including glomerulosclerosis, *Candida* esophageal infection, and cytomegalovirus viremia, the patient was maintained on immunosuppressants. The patient was admitted for right-sided weakness and right-sided facial droop lasting eight hours. The patient’s mentation, speech, reflexes, sensations, and proprioception were all normal. A chest radiograph showed no pulmonary disease, but brain CT and MRI revealed a ring-enhancing hypodense lesion with a local mass effect in her left thalamus, consistent with early abscess formation. No meningeal enhancement was reported, and no lesions were reported in the patient’s spinal cord. CSF analysis showed a nucleated cell with elevated glucose and protein but was negative for several opportunistic infections, including cryptococcal antigen, *Toxoplasma gondii*, and cytomegalovirus; bacterial, acid-fast bacilli and fungal cultures also did not reveal the infection. Although multiple attempts at MALDI-ToF MS identification were unfruitful, microscopic phenotypic analysis accompanied by DNA sequencing of ITS and β-tubulin following a biopsy of the lesion revealed an *A. levis* infection. Despite the presence of an Acrophialophora-related brain abscess, no pulmonary fungal infection was noted.

Differentials

Bacterial Keratitis

The treatment of bacterial keratitis differs significantly from that of fungal keratitis [17]. Due to the similarity in clinical signs, diagnostic microbiology should be used when possible to differentiate between the two microbial causes. The most definitive manner of differentiating between bacterial and fungal keratitis is by culturing the corneal ulcer, then growing and identifying microorganisms. Microscopy may also be used, as the presence of hyphae is sufficient to diagnose fungal keratitis [18]; *Acrophialophora* infection will have corneal scrapings that are positive for septate fungal hyphae [2,12].

Clinical symptoms vary between cases of bacterial and fungal keratitis. A lack of anterior chamber fibrin makes a fungal infection more likely [18]. Satellite lesions, which strongly suggest fungal infections and irregular feathery borders, are associated with filamentous fungi, including *Acrophialophora* [17]. Other factors that favor fungal keratitis include serrated infiltrate margins and a raised surface profile [18].

Examining the circumstances surrounding infection is another valuable tool in differentiating between bacterial keratitis and fungal keratitis, including* Acrophialophora* infection. Fungal keratitis often occurs following a corneal injury involving soil or plant material and should be strongly suspected in these circumstances. Regardless of the diagnosis made, close follow-up is necessary to ensure that the therapy worked and that the cause of infection was not misidentified [19].

Herpes Simplex Keratitis (HSK)

Ocular herpes simplex virus (HSV) infections can trigger corneal, conjunctival, uveal, or retinal disease by inducing inflammation [20]. HSK is the most common of these ocular diseases [21]. The clinical presentations of different HSK subtypes vary, but common symptoms, including watery eyes or discharge, irritation, itching or pain, and light sensitivity, are similar to those of an *Acrophialophora* infection. As also seen in *Acrophialophora* infection, infected eyes may appear whitened, gray-white, or opaque; ulceration may also be present. A typical presentation for epithelial keratitis, the most common HSK subtype, involves the formation of a dendritic corneal ulcer [22] following the integration of numerous punctate lesions [21].

A medical history involving instances of HSK may favor the diagnosis of HSK over *Acrophialophora* infection due to the recurrent nature of the disease. Slit-lamp examination is predominantly used in the diagnosis of HSK [23]. The use of lissamine green or rose bengal dye may assist in visualization, though the use of these dyes may reduce the sensitivity of PCR testing [24]. 

PCR may also be used in HSK diagnosis, though PCR may be less sensitive to infection in patients who use or have used antiviral medications, as well as in patients with atypical presentations [25]. Samples for PCR analysis may be obtained from corneal scrapings or patient tears; though corneal scraping collection may be contraindicated in some circumstances due to reduced corneal thickness from recurrent infection, PCR analysis of tears is less sensitive in detecting the virus [26].

Pyogenic or Tuberculous Abscess

Adequate management of a brain abscess necessitates identifying the causative agent and initiating treatment as soon as possible [27]. *Acrophialophora* brain abscesses typically present as a ring-enhancing lesion with local mass effects [6]. The lesion is hypodense, and thick yellowish pus may be drained from the abscess [1]. The finding of hyphae is indicative of a fungal abscess, and septate hyphae may be found upon examination of aspirated or biopsied material in an *Acrophialophora* fungal abscess. Irregular walls, either lobulated or crenated, are typical of fungal abscesses [27], and irregular rim enhancement may be seen in *Acrophialophora* lesions [13]. A study examining the apparent diffusion coefficient (ADC) of fungal, pyogenic, and tuberculous abscesses found that the wall and projections of fungal abscesses have a low ADC, while the cavity of the abscess has a high ADC [27].

Pyogenic abscesses may feature either smooth or lobulated walls, and both the wall and the cavity of pyogenic abscesses exhibit low ADC [27]. A study examining numerous pyogenic abscesses found a complete hypointense T2 rim in 95.4% of abscesses [28]. Another study indicated that, on diffusion-weighted imaging (DWI), homogenously hyperintense lesions were present in 60% of pyogenic abscess patients, compared to none of the patients with fungal abscesses [29]. PMRS may also be used to differentiate pyogenic from fungal and tuberculous abscesses, as acetate and succinate are only present in pyogenic abscesses [30].

The clinical features of tuberculous abscesses are similar to those of *Acrophialophora*-related fungal abscesses, involving focal neurological deficits, facial weakness, ear discharge, headache, fever, or seizures [29]. A history of pulmonary tuberculosis may or may not be present. Tuberculous brain abscesses may be unilocular or multilocular and have smooth, lobulated, or crenated walls [27,29]. Like *Acrophialophora* lesions, tuberculous abscesses may have a significant mass effect [29]. Differing from the ADC of fungal brain abscesses, tuberculous abscesses have a low ADC in both the wall and cavity. The presence of intracavitary projections favors fungal abscesses over tuberculous abscesses [27].

Histoplasmosis

Histoplasmosis is caused by *Histoplasma capsulatum*, a dimorphic fungus with a saprophytic mold form that grows best in soils associated with bird and bat droppings [31]. Once in the alveoli, the fungus transforms into a yeast morphology [32]. Like *Acrophialophora*, contact with organic material is a risk factor for histoplasmosis since infection occurs following inhalation of aerosolized microconidia [33]. Construction, farming, agriculture, and other activities through which soil or guano contact is established may lead to histoplasmosis [34]. 

Histoplasmosis is often misdiagnosed due to the wide variety of possible clinical manifestations [35]. Although illness in most patients is subclinical, clinical symptoms of disseminated infection include fever, chest pain, cough, other respiratory symptoms, and weight loss; skin and oral lesions may also be present [36]. Due to reduced immune function in AIDS patients, histoplasmosis typically presents as a disseminated infection in this population [31]. Patients with other immunosuppressive disorders, patients who are taking immunosuppressive drugs, infants, transplant recipients, and elderly patients are also likely to exhibit full-body involvement [36-38].

One potential complication of disseminated infection is an ocular disease. In one case study, a patient presented with a two-month history of a gradually increasing painless mass and yellow purulent discharge. Although eye discharges are also commonly seen in *Acrophialophora* infection [12], round yeast bodies consistent with *H. capsulatum* were visualized from excised tissue. The Giemsa stain visualized a bloated microphage with encapsulated bodies inside, allowing for the diagnosis of *H. capsulatum* [39]. 

CNS involvement occurs in 5 to 10 percent of patients with disseminated histoplasmosis [40]. CNS *H. capsulatum* infection may present as a component of disseminated disease or manifest independently of full-body involvement [41]. Clinical features are very similar to those of cerebral *Acrophialophora* infection and include headache, altered mental status, stroke syndromes, confusion, and focal deficits, in addition to the aforementioned symptoms of disseminated infection [35]. The presence of chronic meningitis and spinal cord lesions favors the diagnosis of *H. capsulatum* infection and may allow for clinical differentiation between the two diseases [40,41].

Common radiologic findings in CNS histoplasmosis include focal mass lesions in the brain or spinal cord, diffuse white matter changes, and areas of restricted diffusion [36]. One case study involved a patient who initially had fatigue, weight loss, and cough [35]. A CT scan of the patient’s chest found a mass in his right adrenal gland, but the patient declined a biopsy. Brain imaging revealed multiple lesions, and the patient underwent a lumber puncture and was found to have normal CSF. 

Three months later, the same patient visited a neurologist for neck pain and paresthesia beginning in his right hand and traveling up his arm. Several ring-enhancing lesions were present throughout his brain and spine. A CT with no contrast taken one month later showed multiple hypointense lesions with a hyperintense signal consistent with hemorrhage. An MRI showed multiple ring-enhancing lesions throughout the brain and spinal cord and hyperintense lesions in the spinal cord. Meningeal enhancement was present. A chest CT showed bilateral adrenal masses. At this time, the patient had deteriorated further clinically; one week prior, the patient had a short-term episode of speech difficulty and had an altered mental status at the time of the final scans. A needle biopsy revealed an *H. capsulatum* infection.

The presence of meningeal enhancement on a brain MRI is extremely indicative of a histoplasmosis abscess over *Acrophialophora*. No instances of *Acrophialophora* infection have been reported to involve meningitis. Lesions are also not reported to be present on the spinal cord in *Acrophialophora* infection. Finally, adrenal involvement, including adrenal masses, highly favors histoplasmosis over *Acrophialophora*; adrenal involvement is found in 80 to 90 percent of disseminated histoplasmosis cases [35] but not in any* Acrophialophora* cases. 

Toxoplasmosis

Toxoplasmosis is caused by the protozoan parasite *Toxoplasma gondii* [42] and is often acquired through the ingestion of undercooked meat, feline feces, contaminated water, or contaminated soil. In immunocompetent hosts, toxoplasmosis is asymptomatic in over 80% of instances [43]. Latent infection is present in some infected hosts; *T. gondii* resides in its cystic stage, unaccompanied by any symptoms, but may become reactivated if the patient becomes immunocompromised. This is of particular concern to transplant recipients because cysts from infected transplant organs can also cause disease in previously uninfected recipients [42].

One study describes toxoplasmosis in an acute myeloid leukemia patient who underwent hematopoietic stem cell transplantation (HSCT) [42]. Symptoms were similar to those of a CNS *Acrophialophora* infection: the patient had a fever, new-onset headache and confusion, and a stiff neck. An MRI of the patient’s brain found multiple lesions with multiple peripheral contrast, with the contrast increasing after contrast agent is injected. CSF glucose was normal. Ultimately, CSF toxoplasma DNA was detected positively via PCR test, validating a TE diagnosis.

Depending on the host’s immune function, clinical manifestations and outcomes may vary. Toxoplasma encephalitis (TE) is the most commonly seen infection in AIDS patients and typically manifests as one or more CNS mass lesions [44,45]. TE is generally multifocal but can present nonfocally; over 80 percent of patients will have multiple focal lesions on MRI [46]. TE symptoms can mirror those of an​​​​​​​ *Acrophialophora* CNS infection, making a diagnosis based on symptoms alone difficult. TE patients will present with confusion and may or may not have focal deficits. Focal deficits initially appear transiently and become persistent as the disease progresses. Speech abnormalities and hemiparesis are the most common initial focal findings and may be accompanied by other symptoms, including headache, loss of coordination, lethargy, severe memory loss or dementia, and seizures [46]. 

Toxoplasmosis brain abscesses appear as non-attenuated or low-attenuated lesions on a non-contrast CT [47]. On contrast-enhanced MRI, a target sign may be seen in abscesses; this presentation favors toxoplasmosis brain abscess over *Acrophialophora* infection as the cause of lesions [48]. T1-weighted (T1W) images may show a hypointense or isointense center surrounded by a hyperintense outer layer, while T2-weighted (T2W) images and fluid-attenuated inverted recovery (FLAIR) images show a hyperintense center surrounded by a hypointense region and another hyperintense outer edge [48,49]. Patients may have only the T1W target sign or only the T2W/FLAIR target sign [49]. TE can also be diagnosed quickly and accurately over the ​​​​​​​*Acrophialophora* with a positive PCR result of blood or CSF in combination with cranial imaging. Negative PCR results, however, may not rule out infection [42]. An early biopsy is also recommended to confirm a TE diagnosis.

L. prolificans

*A. fusispora* has been misidentified as *Scedosporium prolificans*, a fungus that is now known as *Lomentospora prolificans* and that has recently been excluded from the Scedosporium genus [11,50]. The diagnosis of *L. prolificans* is typically carried out via analysis of clinical samples [51]. One case study attributed a patient’s fungal keratitis to *L. prolificans* [12]. The causative fungus was later identified as *A. fusispora* based on the conidia being individual as well as in chains. When differentiating between *Acrophialophora* and *L. prolificans*, it is important to note that the ovoid conidia in *L. prolificans* are in slimy heads [10]. Additionally, *L. prolificans* conidiogenous cells are flask-shaped and in a brush-like arrangement, not single on hyphae as in *A. fusispora*.

Finely echinulate conidia ornamentation, often with distinct spiral bands, is a defining feature of *A. fusispora* [10]. Both *A. fusispora* and* L. prolificans* form flask-shaped conidiogenous cells, making differentiation between the two species more challenging. *A. fusispora* exhibits limoniform conidia that are usually arranged in spiral bands and form chains, whereas* L. prolificans* have conidia that are exhibited in slimy heads and are clavate and smooth [11].

In addition to careful morphological analysis, sequencing in the ITS region should be used to confirm whether a fungal species is *Acrophialophora spp*. A lack of molecular diagnostic evidence may lead to inaccurate conclusions about the fungal agent responsible for infection [7].

Diagnosis

No consolidated plan exists for the diagnosis of* Acrophialophora* infection, posing a challenge to clinicians. Due to the rarity of the disease and the scarcity of *Acrophialophora* samples available, the identification of fungal isolates is a challenge. The site of sample collection varies from patient to patient and is dependent upon the apparent site of infection. Corneal scrapings should be taken from patients with keratitis, brain tissue from brain biopsies should be taken from patients with evidence of cerebral infection, bronchoalveolar lavage samples should be taken from patients with CF to assess colonization, and sputum samples should be taken for patients with symptoms of pulmonary infection [6]. 

A diagnosis of *Acrophialophora* infection should be made after careful examination of clinical, radiologic, demographic, and microscopic evidence. Clinical indications outside of the more broad fever, cough, and abscess symptoms include yellow or white sputum expectoration with or without blood [7], frank purulence aspirated from abscesses, and focal neurological deficits [6]. When aspirated and biopsied, grayish and soft necrotic brain tissue with granulomatous inflammation at the site of infection may be visualized in patients with CNS *Acrophialophora* infection [1].

Difficulties in cranial sampling to perform a biopsy are a major contributor to poor clinical outcomes in patients with CNS *Acrophialophora* infection [6]. It is, therefore, essential that imaging signs consistent with *Acrophialophora* infection are known such that *A. fusispora* is retained as a differential in circumstances in which biopsy is contraindicated but radiological signs are consistent with infection. In CNS *Acrophialophora* infection, lesions will appear as hyperintense hypodensities with local mass effect on a non-contrasted CT scan [6,13]. Irregular rim enhancement will be present [13]; lesions will be ring-enhancing on T2 sequencing when an MRI is performed or when a CT with contrast is performed [1,6]. Lesions will be consistent with abscess formation, and numerous peripheral infarcts may also be visible with T2 sequencing on MRI [6]. The lesions will have a mass effect and may have perifocal edema and ventriculitis [13]. Although these ring-enhancing lesions do not independently confirm *Acrophialophora* infection, their presence can validate CNS symptoms. 

In instances of *Acrophialophora* lung infection, a chest CT will show nodular lung lesions with cavitation [1]. Depending on the progression of the disease, lesions may penetrate through the diaphragm. In one patient with emphysema and an *A. levis* infection, a large consolidation, ground-glass opacification, honeycomb formations, a lung balloon formation, and bilateral pleural effusion were present [7]. Bilateral interstitial infiltrates may also be seen in chest X-rays of patients with *Acrophialophora* lung infection [11].

The circumstances surrounding the infection may also provide a strong case for an ​​​​​​​*Acrophialophora* infection. One agricultural worker presented with *A. fusispora* fungal keratitis 1.5 months after an injury involving a wood chip in her eye [11]. Another patient with *A. levis*-induced severe pneumonia had his house demolished for rebuilding and remodeling before illness. Inhalation of dust from the demolished house may have contributed to his infection [7]. If a patient reports contact with or inhalation of organic matter in their daily life or through an injury, an​​​​​​​ *Acrophialophora* infection should be highly suspected. Infection should also be carefully considered in patients who are exposed to agriculture, farming, construction, gardening, or other circumstances through which soil or dust exposure is possible.

Isolation and analysis leading to the identification of *Acrophialophora spp*. is the most definitive method of diagnosis. Genetic confirmation is the gold standard for *Acrophialophora* diagnosis and isolates from all patients suspected of being infected should be tested to verify a positive diagnosis. For genetic confirmation, colonies may be selected following sample collection and plating, and the ITS and β-tubulin regions may be amplified and sequenced [5]. Results can be inputted into NCBI BLAST, and sequence homology to *Acrophialophora* species may be assessed [7]. In the absence of genetic confirmation, however, microscopic histologic findings serve as compelling evidence of *Acrophialophora* infection. 

Nuclear imaging has become increasingly clinically relevant in diagnosing infections, specifically with the use of fluorodeoxyglucose (FDG) radiotracer with positron emission tomography (PET) [52,53]. The utility of PET has expanded far beyond its original oncological purpose to study various conditions, including infectious disease, inflammatory cardiological conditions, neurodegeneration, and nephritis [54-61]. FDG-PET is incredibly specific for detecting granulomatous and nodular inflammation, particularly at the early stages of disease burden. Furthermore, FDG-PET is frequently coregistered with CT to provide anatomical localization. Given the established utility of CT in *Acrophiliophora* infections, adding FDG-PET could further quantify the disease burden, particularly at the very early stages of the disease.

Treatment

Guidelines for the antifungal treatment of *Acrophialophora* infection have not been established; this is due to the scarcity of documented cases and in-vivo testing [1]. Due to the lack of clinical guidelines and differences in antifungal susceptibility across varying *Acrophialophora* strains, testing and treating the cultured strain with the drug having the most potent in-vitro activity may be indicated [5]. Combination antifungal therapy, typically with LAMB and an azole, maybe the most effective [6]. The therapeutic management of *Acrophialophora* infection requires the aggressive use of antifungal agents due to the potential for morbidity and mortality. Long-term antifungal therapy may be required [14]. 

Azole Medications

Voriconazole: *A. fusispora* isolated from corneal scrapings of a patient with mycotic keratitis was found to have a MIC of 0.25 µg/ml for VRC. *A. levis* antifungal susceptibility testing indicated a MIC of 0.25µg/ml. VRC has demonstrated mixed clinical results when used to manage *Acrophialophora* infections. A patient with fungal keratitis secondary to *A. fusispora* infection continued to deteriorate despite treatment with topical VRC, among other medications [2]. Another patient with fungal uveitis improved, however, after treatment with systemic VRC [14]. VRC may be an effective choice in the management of CNS infections due to its activity in both brain tissue and cerebrospinal fluid [6]. A patient with an ​​​​​​​*A. levis-*related brain abscess was treated with VRC, causing her mental status to return to baseline with some neurological symptoms remaining.

Itraconazole: *A. fusispora* isolated from corneal scrapings of a patient with mycotic keratitis was found to have a MIC of 0.25 µg/ml for ITRA [2]. Several isolates of *A. fusispora* were inhibited by relatively low concentrations of ITRA, with MICs ranging from 0.06-0.25 µg/ml [1].

In one case study, systemic ITRA, in combination with other medications, was ineffective in managing fungal keratitis secondary to *A. fusispora* infection [2]. Another patient with pulmonary *A. fusispora* infection showed marked clinical and radiological improvement after three months of treatment with oral ITRA [5]. ITRA may have decreased efficacy in the treatment of *Acrophialophora* CNS involvement due to its limited activity in cerebrospinal fluid [6].

Miconazole: *A. fusispora* isolates were found to be inhibited by relatively low concentrations of miconazole, with the MIC of miconazole ranging from ≤0.03-0.06 μg/ml across the samples [1]. In-vitro testing of *A. fusispora* isolates causing mycotic keratitis showed that *A. fusispora* was more sensitive to miconazole than clotrimazole, AMB, and lactones. In-vivo testing of the same isolate in rabbits, however, indicated that AMB was more effective than miconazole in alleviating infection [3].

Posaconazole: *A. fusispora* isolated from corneal scrapings of a patient with mycotic keratitis was found to have a MIC of 0.125 µg/ml for posaconazole [2]. Antifungal susceptibility testing for *A. levis* obtained from a patient with a brain abscess indicated a MIC of 0.06 µg/ml for posaconazole.

Fluconazole: In general, fluconazole (FLU) is less effective as an antifungal agent against opportunistic filamentous fungi [1]. Antifungal susceptibility tests conducted against several isolates of *A. fusispora* indicated that* A. fusispora* demonstrated dose-dependent susceptibility to FLU and was only susceptible to relatively high concentrations. The MIC of FLU for the isolates tested ranged from 8-64 μg/ml. Another study analyzing *A. fusispora* isolated from the corneal scrapings of a patient with mycotic keratitis found that the MIC of FLU was 4 µg/ml [2]. Antifungal susceptibility testing for *A. levis* obtained from a patient with a brain abscess showed a MIC of 8μg/ml FLU [6].

Amphotericin B

The MIC of AMB ranged from 0.25 to 2 μg/ml when several *A. fusispora* isolates were assessed. Minimum lethal concentrations (MLC) ranged from 1.0-16 μg/ml [1]. Antifungal susceptibility testing of *A. levis* obtained from a brain abscess patient indicated a MIC of 1.5 μg/ml for AMB [6].

Although higher AMB concentrations may not be achievable safely in plasma, it was found that *A. fusispora* isolates obtained from humans were more susceptible to AMB than nonhuman isolates, with lower AMB MLCs than nonhuman isolates [1]. Sufficient drug concentrations may, therefore, be safely achieved in-vitro depending on the nature of the strain being treated. The high AMB MICs and MLCs observed for some strains of *A. fusispora* indicate, however, that antifungal therapy with conventional or lipid AMB may fail.

In-vivo analysis of experimental *A. fusispora* infection in rabbit corneal lesions found that AMB exhibited better therapeutic results than miconazole, tolciclate, clotrimazole, and lactones [3]. One patient who was treated with AMB for pulmonary *A. fusispora* infection had an increase in the size and number of pulmonary nodules after three weeks of treatment [1]. The patient also showed no improvement after being treated with LAMB for 26 days and began to exhibit new neurological symptoms, indicating possible disease progression.

AMB and LAMB treatment has caused the worsening of symptoms in several patients with *Acrophialophora* infection [14]. For one patient with conjunctival ulcer and uveitis, LAMB was prescribed following systemic ITRA, but the patient was switched from LAMB to systemic and topical VRC because the patient’s symptoms worsened. Symptoms stabilized after the patient was taken off of AMB.

Another patient with an ocular *A. fusispora* infection was treated with several antifungal medications. Treatment with AMB was attempted but caused flares and was withdrawn [2].

Natamycin

Antifungal susceptibility tests of one *A. fusispora* fungal keratitis isolate indicated a relatively high MIC of 4 μg/ml for natamycin [2]. Clinically, a fungal keratitis patient treated with natamycin eye drops, among other antifungal treatments, showed no improvement, with the patient’s vision continuing to deteriorate.

Potassium Iodide

One case study involving *A. fusispora*-related keratitis reports that the patient recovered after treatment with potassium iodide in combination with nystatin eye ointment, atropine, and antibiotic eye drops to treat a bacterial coinfection [3].

Anti-Inflammatory Agents

Steroid medications may be used to treat respiratory fungal infections before their fungal cause is known. Corticosteroid treatment, however, is associated with an increased fungal load in pulmonary fungal infections [30]. Furthermore, it is known that immunocompromised patients are at increased risk for severe *Acrophialophora *infections and complications. The use of steroid medications in the clinical management of *Acrophialophora* infection may therefore contribute to morbidity and mortality.

In an experiment comparing ocular *A. fusispora* infections in immunocompetent and immunocompromised rabbits, experimental infections were more severe in rabbits pretreated with cortisone [3].

One case study describes a patient with AIDS who was treated for cryptogenic organizing pneumonia with corticosteroids. The patient’s lung condition improved with prednisolone treatment but worsened when the corticosteroid dose was reduced. After four months of corticosteroid therapy and a bowel resection surgery, the patient was admitted for fever, headache, and an altered level of consciousness [13]. The patient was later found to have a fatal *A. fusispora* brain abscess, indicating the importance of monitoring patients who are immunocompromised or on immunosuppressive drugs for invasive fungal infections.

Surgical Intervention

Based on several case studies, a combination of surgical intervention and antifungal treatment may be effective in treating* Acrophialophora* infection [6]. In one case study involving keratouveitis secondary to *A. fusispora* infection, surgical debridement was effective in restoring the patient’s eyesight and relieving inflammation [9,10,12]. Infected tissue was limited to the area immediately surrounding a lost contact lens in the eye [12].

Guidelines for Treatment

Adequate therapeutic management of *Acrophialophora* infection must involve rapid and aggressive treatment with antifungal agents due to the pathogenicity of the fungi. As soon as fungal infection is suspected, culture and sensitivity tests should be carried out to determine the etiologic agent and determine a treatment course. Since varying *Acrophialophora* isolates have varying susceptibilities to different antifungals and because clinical studies exhibit mixed results for the efficacy of many antifungal drugs, performing antifungal sensitivity assays should be a priority in determining a course of treatment [1].

In-vitro sensitivity assays may be carried out via colorimetric microdilution [7] or broth microdilution [6]. Antifungal drug MICs and MLCs that are ascertained during in-vitro testing for the isolate should be used to decide on which antifungal treatment to pursue [7]. In-vivo susceptibility of isolates to medications should be frequently assessed throughout treatment through the investigation and monitoring of clinical and radiological signs.

*Acrophialophora* infection is susceptible to combination antifungal therapy; combining AMB and azole medication has demonstrated clinical efficacy in treating *Acrophialophora* infection [1,6]. If CNS abscess or disseminated infection is suspected, surgical resection of the abscess should be performed, and combination antifungal therapy should be initiated [6]. If antifungal susceptibility is not known, VRC may be valuable for patients with CNS infection due to its penetration into the brain and CSF. ITRA has also had some instances of success in cerebral *Acrophialophora* case studies but has low penetration into the CSF.

*Acrophialophora* eye infections vary in severity; if the ocular infection is localized, surgical debridement may achieve favorable results [12]. Due to the high prevalence of perforated corneal ulcers in patients with​​​​​​​ *Acrophialophora* fungal keratitis, topical and systemic antifungal agents should also be prescribed according to the results of antifungal susceptibility tests [2]. Antifungal treatment has demonstrated mixed efficacy in patients with *Acrophialophora* eye infections, and therapeutic keratoplasty is often necessary following antifungal treatment failure.

When treating *Acrophialophora *infection, note that granulocyte colony-stimulating factor may be useful as a supplemental treatment in immunocompromised patients, as could reducing an immunocompromised patient’s steroid dose [1]. AMB and LAMB have caused flares when used in the treatment of *Acrophialophora* infection [1,7]. If AMB or LAMB neglects to halt disease progression, the dose may need to be increased based on antifungal susceptibility. If flares occur, the medication should be discontinued.

In the long-term management of *Acrophialophora* infection, suppressive therapy should be used over a period of several months to enhance remission and prevent relapse or complications [1]. Follow-up should be conducted during remission to monitor for clinical or radiological signs of relapse or spread.

## Conclusions

*Acrophialophora* is an opportunistic pathogen capable of producing a range of clinical manifestationsin humans, including fungal keratitis, pulmonary infection, and brain abscess. Infection by *Acrophialophora* or other opportunistic fungi can be life-threatening and is of particular concern in immunocompromised patients, who typically present with more severe symptoms and systemic infection. It is clear that an *Acrophialophora* infection requires aggressive treatment to reduce morbidity and mortality. Early identification of the pathogen can help to minimize the progression of the disease, prevent mortality, and reduce instances of permanent eye, lung, and brain damage.

Barriers to timely diagnosis and treatment of *Acrophialophora* infection include failure to rule out other bacterial and viral causes of disease, misidentification of the causative fungi, the potential absence of classical symptoms in immunocompromised patients, and a lack of standardized treatment guidelines. Future research should involve the collection of antifungal susceptibility data and the development of standardized therapeutic regimens to prevent disease progression. In clinical practice, guidelines for the diagnosis of *Acrophialophora* should be employed as soon as a fungal infection is suspected to reduce instances of mortality. Finally, immunocompromised patients, patients with COPD, and patients receiving immunosuppressive therapies should be closely monitored for the development of opportunistic fungal infections, including *Acrophialophora*.

## References

[1] Al-Mohsen IZ, Sutton DA, Sigler L (2000). Acrophialophora fusispora brain abscess in a child with acute lymphoblastic leukemia: review of cases and taxonomy. J Clin Microbiol.

[2] Sharma S, Singla N, Gulati N, Arya SK, Chander J (2023). Acrophialophora fusispora as an agent of mycotic keratitits: A case report and review of literature. Infect Disord Drug Targets.

[3] Shukla PK, Khan ZA, Lal B, Agrawal PK, Srivastava OP (1983). Clinical and experimental keratitis caused by the Colletotrichum state of Glomerella cingulata and Acrophialophora fusispora. Sabouraudia.

[4] Cimon B, Challier S, Béguin H, Carrère J, Chabasse D, Bouchara JP (2005). Airway colonization by Acrophialophora fusispora in patients with cystic fibrosis. J Clin Microbiol.

[5] Samaddar A, Sharma A, Shrimali T (2020). Pulmonary infection due to Acrophialophora fusispora in a patient with underlying mixed connective tissue disease and chronic pulmonary aspergillosis: A case report and review of literature. J Mycol Med.

[6] Modlin CE, Collins LF, Burd EM, Lockhart SR, Marshall Lyon G (2020). Acrophialophora levis brain abscess in a kidney transplant patient: A case report and review of the literature. Med Mycol Case Rep.

[7] Huang J, Liu Z (2019). The first case of Acrophialophora levis-induced severe pneumonia: a case report and literature review. BMC Infect Dis.

[8] González-Escalada A, Del Palacio A, Calvo MT, Gené J, Guarro J (2000). [Two cases of scalp wound colonization and respiratory tract by mycelial fungi]. Rev Iberoam Micol.

[9] Sandoval-Denis M, Gené J, Sutton DA, Wiederhold NP, Guarro J (2015). Acrophialophora, a poorly known fungus with clinical significance. J Clin Microbiol.

[10] Guarro J, Gené J (2002). Acrophialophora fusispora misidentified as Scedosporium prolificans. J Clin Microbiol.

[11] Guarro J, Mendiratta DK, De Sequeira H (2007). Acrophialophora fusispora: an emerging agent of human mycoses. A report of 3 new clinical cases. Diagn Microbiol Infect Dis.

[12] Arthur S, Steed LL, Apple DJ, Peng Q, Howard G, Escobar-Gomez M (2001). Scedosporium prolificans keratouveitis in association with a contact lens retained intraocularly over a long term. J Clin Microbiol.

[13] Li CW, Lee HC, Chang TC (2013). Acrophialophora fusispora brain abscess in a patient with acquired immunodeficiency syndrome: a case report and review of the literature. Diagn Microbiol Infect Dis.

[14] Watanabe Y, Kobayashi T, Nakamura I (2018). A case of conjunctival ulcer and uveitis caused by Acrophialophora sp. in an immunocompromised patient: a case report and riterature review. Jpn J Infect Dis.

[15] Zhang Y, Liu F, Wu W, Cai L (2015). A phylogenetic assessment and taxonomic revision of the thermotolerant hyphomycete genera Acrophialophora and Taifanglania. Mycologia.

[16] Groll AH, Piscitelli SC, Walsh TJ (1998). Clinical pharmacology of systemic antifungal agents: a comprehensive review of agents in clinical use, current investigational compounds, and putative targets for antifungal drug development. Adv Pharmacol.

[17] Dalmon C, Porco TC, Lietman TM (2012). The clinical differentiation of bacterial and fungal keratitis: a photographic survey. Invest Ophthalmol Vis Sci.

[18] Leck A, Burton M (2015). Distinguishing fungal and bacterial keratitis on clinical signs. Community Eye Health.

[19] Rodman RC, Spisak S, Sugar A, Meyer RF, Soong HK, Musch DC (1997). The utility of culturing corneal ulcers in a tertiary referral center versus a general ophthalmology clinic. Ophthalmology.

[20] Lappin M, Wotman K, Chow L, Williams M, Hawley J, Dow S (2023). Nanoparticle ocular immunotherapy for herpesvirus surface eye infections evaluated in cat infection model. PLoS One.

[21] Azher TN, Yin XT, Tajfirouz D, Huang AJ, Stuart PM (2017). Herpes simplex keratitis: challenges in diagnosis and clinical management. Clin Ophthalmol.

[22] Nie AQ, Chen XM, Li Q (2022). Herpes simplex keratitis following Smart Pulse Technology assisted transepithelial photorefractive keratectomy: a case report. BMC Ophthalmol.

[23] Poon SH, Wong WH, Lo AC (2021). A systematic review on advances in diagnostics for herpes simplex keratitis. Surv Ophthalmol.

[24] Seitzman GD, Cevallos V, Margolis TP (2006). Rose bengal and lissamine green inhibit detection of herpes simplex virus by PCR. Am J Ophthalmol.

[25] Kowalski RP, Gordon YJ, Romanowski EG, Araullo-Cruz T, Kinchington PR (1993). A comparison of enzyme immunoassay and polymerase chain reaction with the clinical examination for diagnosing ocular herpetic disease. Ophthalmology.

[26] Satpathy G, Mishra AK, Tandon R (2011). Evaluation of tear samples for Herpes Simplex Virus 1 (HSV) detection in suspected cases of viral keratitis using PCR assay and conventional laboratory diagnostic tools. Br J Ophthalmol.

[27] Luthra G, Parihar A, Nath K (2007). Comparative evaluation of fungal, tubercular, and pyogenic brain abscesses with conventional and diffusion MR imaging and proton MR spectroscopy. AJNR Am J Neuroradiol.

[28] Panyaping T, Sananmuang T, Suriyajakryuththana W (2018). Comparative evaluation of fungal, tuberculous and pyogenic brain abscess with conventional, diffusion, and susceptibility-weighted MR sequences [SWMRS]. J Med Assoc Thai.

[29] Kumar R, Pandey CK, Bose N, Sahay S (2002). Tuberculous brain abscess: clinical presentation, pathophysiology and treatment (in children). Childs Nerv Syst.

[30] Fraczek MG, Chishimba L, Niven RM (2018). Corticosteroid treatment is associated with increased filamentous fungal burden in allergic fungal disease. J Allergy Clin Immunol.

[31] Myint T, Leedy N, Villacorta Cari E, Wheat LJ (2020). HIV-associated histoplasmosis: current perspectives. HIV AIDS (Auckl).

[32] Mittal J, Ponce MG, Gendlina I, Nosanchuk JD (2019). Histoplasma capsulatum: mechanisms for pathogenesis. Curr Top Microbiol Immunol.

[33] PR JJ, PA MI, LO CG (1960). Early pathogenesis of experimental histoplasmosis. Arch Pathol.

[34] McKinsey DS, Spiegel RA, Hutwagner L (1997). Prospective study of histoplasmosis in patients infected with human immunodeficiency virus: incidence, risk factors, and pathophysiology. Clin Infect Dis.

[35] Gonzalez HH, Rane M, Cioci A, Goodman S, Espinosa PS (2019). Disseminated central nervous system histoplasmosis: a case report. Cureus.

[36] Chang B, Saleh T, Wales C (2022). Case report: Disseminated histoplasmosis in a renal transplant recipient from a non-endemic region. Front Pediatr.

[37] Dogenski LC, Pasqualotto EM, Dutra MJ, Rovani G, Trentin MS, De Carli JP (2022). Uncommon case of histoplasmosis with oral manifestation: A case report of diagnosis in a South American patient. Int J Surg Case Rep.

[38] Odio CM, Navarrete M, Carrillo JM, Mora L, Carranza A (1999). Disseminated histoplasmosis in infants. Pediatr Infect Dis J.

[39] Behera RK, Gupta PC, Khurana S, Sehgal S, Sharma S, Ram J (2022). A rare presentation of ocular histoplasmosis in a patient with systemic nocardiosis. Indian J Ophthalmol.

[40] Wheat J, Myint T, Guo Y (2018). Central nervous system histoplasmosis: Multicenter retrospective study on clinical features, diagnostic approach and outcome of treatment. Medicine (Baltimore).

[41] Wheat LJ, Musial CE, Jenny-Avital E (2005). Diagnosis and management of central nervous system histoplasmosis. Clin Infect Dis.

[42] Cetinkaya FI, Mumcu N, Unuvar GK, Kilic AU, Kaynar L (2022). Toxoplasmic encephalitis after allogeneic hematopoietic stem cell transplantation. North Clin Istanb.

[43] Robert-Gangneux F, Dardé ML (2012). Epidemiology of and diagnostic strategies for toxoplasmosis. Clin Microbiol Rev.

[44] Giovane RA, Lavender PD (2018). Central nervous system infections. Prim Care.

[45] Rao M, Subramanya H, Bhardwaj JR, Gupta RM, Ohri VC (1997). Toxoplasma encephalitis (TE): a report on three fatal cases. Med J Armed Forces India.

[46] Luft BJ, Remington JS (1992). Toxoplasmic encephalitis in AIDS. Clin Infect Dis.

[47] Abdel Razek AA, Watcharakorn A, Castillo M (2011). Parasitic diseases of the central nervous system. Neuroimaging Clin N Am.

[48] Harrison WT, Hulette C (2017). Cerebral toxoplasmosis: a case report with correlation of radiographic imaging, surgical pathology, and autopsy findings. Acad Forensic Pathol.

[49] Masamed R, Meleis A, Lee EW, Hathout GM (2009). Cerebral toxoplasmosis: case review and description of a new imaging sign. Clin Radiol.

[50] Konsoula A, Tsioutis C, Markaki I, Papadakis M, Agouridis AP, Spernovasilis N (2022). Lomentospora prolificans: An Emerging Opportunistic Fungal Pathogen. Microorganisms.

[51] Ramirez-Garcia A, Pellon A, Rementeria A (2018). Scedosporium and Lomentospora: an updated overview of underrated opportunists. Med Mycol.

[52] Karambelkar P, Rojulpote C, Borja AJ, Youngs C, Bhattaru A (2020). An unusual case of tricuspid valve infective endocarditis caused by Erysipelothrix rhusiopathiae. Cureus.

[53] Ten Hove D, Slart RH, Sinha B, Glaudemans AW, Budde RP (2021). 18F-FDG PET/CT in infective endocarditis: indications and approaches for standardization. Curr Cardiol Rep.

[54] Bhattaru A, Rojulpote C, Ghorpade R (2020). Abstract MP24: correlation between blood pressure and inflammation in the thoracic aorta of HIV patients with and without cocaine use as assessed by FDG-PET/CT. Hypertension.

[55] Bhattaru A, Borja A, Zhang V, Rojulpote KV, Werner T, Alavi A, Revheim M-E (2020). FDG-PET/CT as the superior imaging modality for inflammatory bowel disease. J Nucl Med.

[56] Rojulpote C, Bhattaru A, Jean C (2022). Effect of immunosuppressive therapy and biopsy status in monitoring therapy response in suspected cardiac sarcoidosis. JACC Cardiovasc Imaging.

[57] Borja AJ, Hancin EC, Zhang V (2021). Global brain glucose uptake on 18F-FDG-PET/CT is influenced by chronic cardiovascular risk. Nucl Med Commun.

[58] Rojulpote C, Bhattaru A, Patil S (2022). Abstract 11404: phenotyping cardiac sarcoidosis with PET/MR: imaging characteristics, treatment response, and outcomes of isolated cardiac sarcoidosis versus extra-cardiac sarcoidosis with cardiac involvement. Circulation.

[59] Bhattaru A, Rojulpote C, Gonuguntla K (2021). An understanding of the atherosclerotic molecular calcific heterogeneity between coronary, upper limb, abdominal, and lower extremity arteries as assessed by NaF PET/CT. Am J Nucl Med Mol Imaging.

[60] Borja AJ, Bhattaru A, Rojulpote C (2020). Association between atherosclerotic cardiovascular disease risk score estimated by pooled cohort equation and coronary plaque burden as assessed by NaF-PET/CT. Am J Nucl Med Mol Imaging.

[61] Patil S, Rojulpote C, Gonuguntla K (2020). Association of triglyceride to high density lipoprotein ratio with global cardiac microcalcification to evaluate subclinical coronary atherosclerosis in non-diabetic individuals. Am J Cardiovasc Dis.

